# A New Angle on Transcranial Magnetic Stimulation Coil Orientation: A Targeted Narrative Review

**DOI:** 10.1016/j.bpsc.2024.04.018

**Published:** 2024-05-08

**Authors:** Andris Cerins, Elizabeth H.X. Thomas, Tracy Barbour, Joseph J. Taylor, Shan H. Siddiqi, Nicholas Trapp, Alexander McGirr, Kevin A. Caulfield, Joshua C. Brown, Leo Chen

**Affiliations:** Department of Psychiatry, School of Translational Medicine, Monash University, Melbourne, Victoria, Australia; Department of Psychiatry, School of Translational Medicine, Monash University, Melbourne, Victoria, Australia; Massachusetts General Hospital, Department of Psychiatry, Harvard Medical School, Boston, Massachusetts; Center for Brain Circuit Therapeutics, Brigham and Women’s Hospital, Harvard Medical School, Boston, Massachusetts; Department of Psychiatry, Brigham and Women’s Hospital, Harvard Medical School, Boston, Massachusetts; Center for Brain Circuit Therapeutics, Brigham and Women’s Hospital, Harvard Medical School, Boston, Massachusetts; Department of Psychiatry, Brigham and Women’s Hospital, Harvard Medical School, Boston, Massachusetts; University of Iowa, Department of Psychiatry, Carver College of Medicine, Iowa City, Iowa; Iowa Neuroscience Institute, Iowa City, Iowa; Department of Psychiatry, University of Calgary, Alberta, Canada; Hotchkiss Brain Institute, University of Calgary, Calgary, Alberta, Canada; Brain Stimulation Division, Department of Psychiatry, Medical University of South Carolina, Charleston, South Carolina; Brain Stimulation Mechanisms Laboratory, Division of Depression and Anxiety Disorders, McLean Hospital, Belmont, Massachusetts; Department of Psychiatry, Harvard Medical School, Boston, Massachusetts; Department of Psychiatry, School of Translational Medicine, Monash University, Melbourne, Victoria, Australia; Alfred Mental and Addiction Health, Alfred Health, Melbourne, Victoria, Australia

## Abstract

Transcranial magnetic stimulation (TMS) is used to treat several neuropsychiatric disorders including depression, where it is effective in approximately one half of patients for whom pharmacological approaches have failed. Treatment response is related to stimulation parameters such as the stimulation frequency, pattern, intensity, location, total number of pulses and sessions applied, and target brain network engagement. One critical but underexplored component of the stimulation procedure is the orientation or yaw angle of the commonly used figure-of-eight TMS coil, which is known to impact neuronal response to TMS. However, coil orientation has remained largely unchanged since TMS was first used to treat depression and continues to be based on motor cortex anatomy, which may not be optimal for the dorsolateral prefrontal cortex treatment site. In this targeted narrative review, we evaluate experimental, clinical, and computational evidence indicating that optimizing coil orientation may improve TMS treatment outcomes. The properties of the electric field induced by TMS, the changes to this field caused by the differing conductivities of head tissues, and the interaction between coil orientation and the underlying cortical anatomy are summarized. We describe evidence that the magnitude and site of cortical activation, surrogate markers of TMS dosing and brain network targeting considered central in clinical response to TMS, are influenced by coil orientation. We suggest that coil orientation should be considered when applying therapeutic TMS and propose several approaches to optimizing this potentially important treatment parameter.

Transcranial magnetic stimulation (TMS) is a noninvasive brain stimulation technique. A TMS device sends a momentary current through an insulated conductive coil held against the scalp, generating a brief and time-varying magnetic field, which induces a directional electric field (E-field) in the underlying cortical tissue that is capable of modulating neural activity (1). The E-field induced in the cortex is greater in closer proximity to the coil and attenuates rapidly with distance (2,3). E-fields of sufficient magnitude can elicit action potentials in cortical neurons, primarily in superior gray matter (GM) (4–7). The TMS E-field is influenced by the shape of the coil, and different coil designs can induce E-fields of varying depth and spread [see details in Deng *et al*. (8) and Drakaki *et al*. (9)]. Here, we focus on the directional stimulation achieved by the figure-of-eight design (10) that is widely used in clinical and research settings.

Repetitive TMS (rTMS) protocols have been shown to alter brain activity beyond the period of initial administration (11,12). This ability to safely and noninvasively induce changes in the human brain is valuable in neurophysiological and clinical research. As a therapeutic modality, rTMS is U.S. Food and Drug Administration cleared for treatment-resistant major depressive disorder and several other neuropsychiatric conditions (13). However, interindividual and intertrial response to rTMS treatment for major depressive disorder is variable, with recent large registry studies reporting intention-to-treat response and remission rates of 42% to 58% and 16% to 28%, respectively (14,15). While it is beneficial for many patients, identifying ways to improve clinical outcomes is a high priority. To this end, there is an increasing interest in improving rTMS treatment outcomes by optimizing parameters such as stimulation intensity or cortical stimulation site (16,17). Recent preliminary studies that have implemented individualized stimulation targets and personalized stimulation intensities have reported greatly improved therapeutic efficacy, achieving response rates of 85% to 90% (18,19). However, other parameters were concurrently adjusted in these studies, such as the total number of pulses delivered, which makes it difficult to determine how each adjustment contributed or interacted to produce these remarkable response rates (16,20).

Another parameter, the orientation, i.e., yaw angle (Figure 1) of the TMS treatment coil, interacts with the underlying cortical anatomy to influence the intensity and location of TMS treatment (21,22). However, in the drive to improve response to rTMS, coil orientation has largely remained overlooked, and little research has focused on methods for optimizing this treatment parameter. This narrative review focuses on the influence of TMS coil orientation on the magnitude and location of E-fields and how coil orientation might be optimized to improve therapeutic outcomes.

## THE TMS E-FIELD

The first TMS coils were annular and produced a corresponding and evenly distributed circular E-field (8). The figure-of-eight coil comprises 2 adjacent circular coil windings (Figure 2). Oppositely directed currents in each coil travel in the same direction at the intersection of the windings (Figure 2), approximately doubling E-field strength near the center of the coil (10) (Figure 3). This concentration of the E-field at the figure-of-eight coil center can be used for targeted stimulation of specific cortical areas. Here, the area under the coil where the focal E-field magnitude is more than half of the maximum E-field value is approximately 5 cm^2^ and reaches typical depths of 1 to 3.5 cm (8).

The concentrated E-field produced at the center of the figure-of-eight coil is directed tangential to the coil windings and runs parallel to the short axis of the coil. Thus, E-field direction is dictated by the yaw angle of the TMS coil, referred to here as current direction or coil orientation. By Lenz’s law, the initial current induced in the cortex flows in the opposite direction to the initial current in the TMS coil windings (Figure 4) (23). The stimulating current in the cortex is effectively unidirectional when TMS pulses are delivered with a monophasic waveform, while biphasic stimulation induces sequential currents in opposite directions (Figure 5).

### Influence of Tissue Types on the E-Field

In a medium with homogeneous electrical conductivity, the E-field is maximal directly below the center of the figure-of-eight coil (10) and reduces with increasing distance from the coil. As such, when TMS is applied to the scalp, E-field magnitude is greater in gyral crowns partly because they are anatomically closest to the coil. However, head tissues have differing electrical conductivities that influence both the magnitude and the location of the peak E-field in cortical tissues (22). A basic mechanism of this influence is that electrical current will preferentially flow via the path of least resistance. Here, the cerebrospinal fluid (CSF) present in sulci and over gyral surfaces provides the easiest path of travel for TMS-induced current because CSF is more conductive than neural tissue (24,25).

Currently, the most widely utilized noninvasive method of evaluating TMS-induced cortical currents is E-field modeling, which uses structural magnetic resonance imaging (MRI)–derived tissue segmentation to account for the differing spatial distribution and electrical conductivity of head tissues when estimating TMS-induced E-fields in the brain (21). Here, using realistic and complex head models is thought to provide more accurate E-field estimates than the previous simple spherical models of the human head. While E-field modeling is theoretical in nature, validation studies have reported that computed TMS E-field magnitude correlates strongly with the amplitude of TMS-elicited physiological motor response (26) and that mapped E-field magnitudes closely align with gold-standard motor mapping conducted with direct electrical stimulation (27).

### Dose: Coil Orientation’s Interaction With Gyral Anatomy Can Alter E-Field Strength

E-field modeling in realistic head tissues has highlighted the impact of local gyral anatomy on the likely site and magnitude of TMS-induced cortical currents (6,21,22,28). This modeling suggests that TMS elicits maximal current in the cortex when the current direction is perpendicular to the local sulcal/gyral anatomy (21,22). The greater cortical E-fields achieved when TMS-induced current is approximately perpendicular to gyri are driven by a boundary effect that occurs when current flows perpendicular to the CSF-GM interface (29). Here, stronger current carried by more conductive CSF enters the GM, producing greater E-field magnitudes in this less conductive tissue (28). The models suggest that directing current perpendicular to gyral crowns increases E-field magnitudes by up to 50% (21,22). However, where current direction is parallel to sulcal/gyral anatomy, this amplification effect is not observed (22). See Figure 6A for an illustration of the effect in stereotyped parallel gyri. This phenomenon has been utilized in motor and visual cortex TMS, where responses are most easily evoked by orienting the TMS coil perpendicular to the major sulci in these regions (30,31). However, this phenomenon is perhaps less well appreciated in therapeutic contexts when rTMS is applied over cortical regions with greater interindividual variation in anatomy. Recent clinical findings that greater E-field magnitudes may be associated with improved response to rTMS treatment of major depressive disorder are relevant to this review (32,33).

### Target: Coil Orientation’s Interaction With Gyral Anatomy Can Alter Peak E-Field Location

In the stereotyped gyri and sulci illustrated in Figure 6A, maximal E-fields in GM occur directly under the center of the coil, partly because the coil center is positioned over a gyral peak. If the coil were centralized over a sulcus, equal maximal E-fields might occur in the 2 flanking gyral crowns. However, when TMS is applied over undulating human gyral anatomy, E-field modeling reveals that the site of maximal activation may not be directly under the center of the coil, largely because the locations where gyral crowns run perpendicular to the TMS-induced current may not be directly under the coil center (21,22). Consequently, the site of the maximal E-field may be laterally displaced from the TMS target identified on the scalp (22,34). Estimates of this lateral displacement vary. An early study reported displacements of 4 to 57 mm, averaging 16.5 mm (22), while later studies reported displacements ranging between 0 and 14 mm (35,36) and average displacements of 5.5 and 10 mm (34,35) (see Figure 6B for illustration). In effect, depending on the coil’s orientation relative to local anatomy, the site of stimulation may be somewhat distant from the center of the coil (22).

### Network: Does Coil Orientation Influence Network Targeting?

The cortical regions that can be reached using TMS are topographically divided into multiple distinct regions that belong to different brain networks (37). Although the mechanisms that underlie the therapeutic efficacy of rTMS have not been fully elucidated, rTMS treatment targets in the dorsolateral prefrontal cortex (DLPFC) are sometimes conceptualized as a node in a distributed brain network where dysfunction contributes to depressive symptoms (38,39). This network hypothesis continues to drive functional connectivity research aimed at optimizing or even personalizing cortical targets for rTMS treatment (40,41). However, coil orientation has largely been ignored when employing these targeting approaches. In fact, coil orientation may impact which network or networks are preferentially modulated by TMS pulses. In clinical practice, coil orientation is commonly determined using 2 approaches. One method aligns the short axis of the coil in a broadly parasagittal direction, often rotated slightly toward the tip of the patient’s nose (42–44), and the other method orients the coil at 45° to the midsagittal plane (45–47), as is commonly implemented in motor cortex TMS. As with other aspects of early therapeutic TMS research, these parameters may have evolved pragmatically, perhaps based on existing practice in other cortical regions, patient tolerability, equipment design, and operator ergonomics. These coil orientations have remained largely unchanged for over 2 decades, suggesting a relatively unexplored avenue that may be relevant for optimization of neuronal stimulation and, in turn, therapeutic effects.

TMS E-field modeling suggests that changes to coil orientation at a fixed scalp position can alter the site of cortical stimulation (21,22), and these different stimulation sites may belong to different networks. Therefore, in theory, changes in coil orientation may alter network activation. Interestingly, computational research has suggested that TMS targeting of brain networks may be coil orientation dependent (48,49), and one model suggested distinct network connectivity profiles and coil orientation dependencies across differing DLPFC targets (48). In this model, more medial and posterior sites, approximately corresponding with the earliest U.S. Food and Drug Administration–approved treatment targets 5 to 6 cm anterior from motor cortex hand representation, primarily targeted the default mode network (DMN), and DMN targeting was maximal when the coil was oriented at 45° to the sagittal plane (48). In contrast, more frontal and lateral sites, approximately corresponding with recently recommended treatment locations (50–52), targeted the frontoparietal network (FPN), and FPN targeting was maximal when the coil was oriented along a parasagittal plane. Importantly, modeled network targeting depended on coil orientation in the region between these 2 sites, suggesting that specific coil orientations could be used to target a specific brain network (Figure 7) (48).

### The TMS E-Field and Cortical Microcircuits

The direction of the TMS E-field can influence cortical microcircuits and may interact with micro-level axonal orientation within the cortical layers (53–55). Therefore, the TMS E-field simultaneously interacts with both macro- and microanatomy. Although the clinical relevance of cell-level sensitivity to TMS current direction requires further investigation, we briefly summarize potentially relevant findings below. In the motor cortex, altering TMS coil orientation, including by 180°, can alter the composition of the descending corticospinal volley and the amplitude and latency of the resultant motor response (56,57). Accumulating evidence suggests that these differences may result from direction-dependent recruitment of distinct neural populations (54,58). These directional sensitivities may be clinically relevant because they have been shown to impact rTMS-induced change in motor plasticity (59) and task performance (60) and influence response to rTMS pain therapy (61,62).

**Figure 1.**
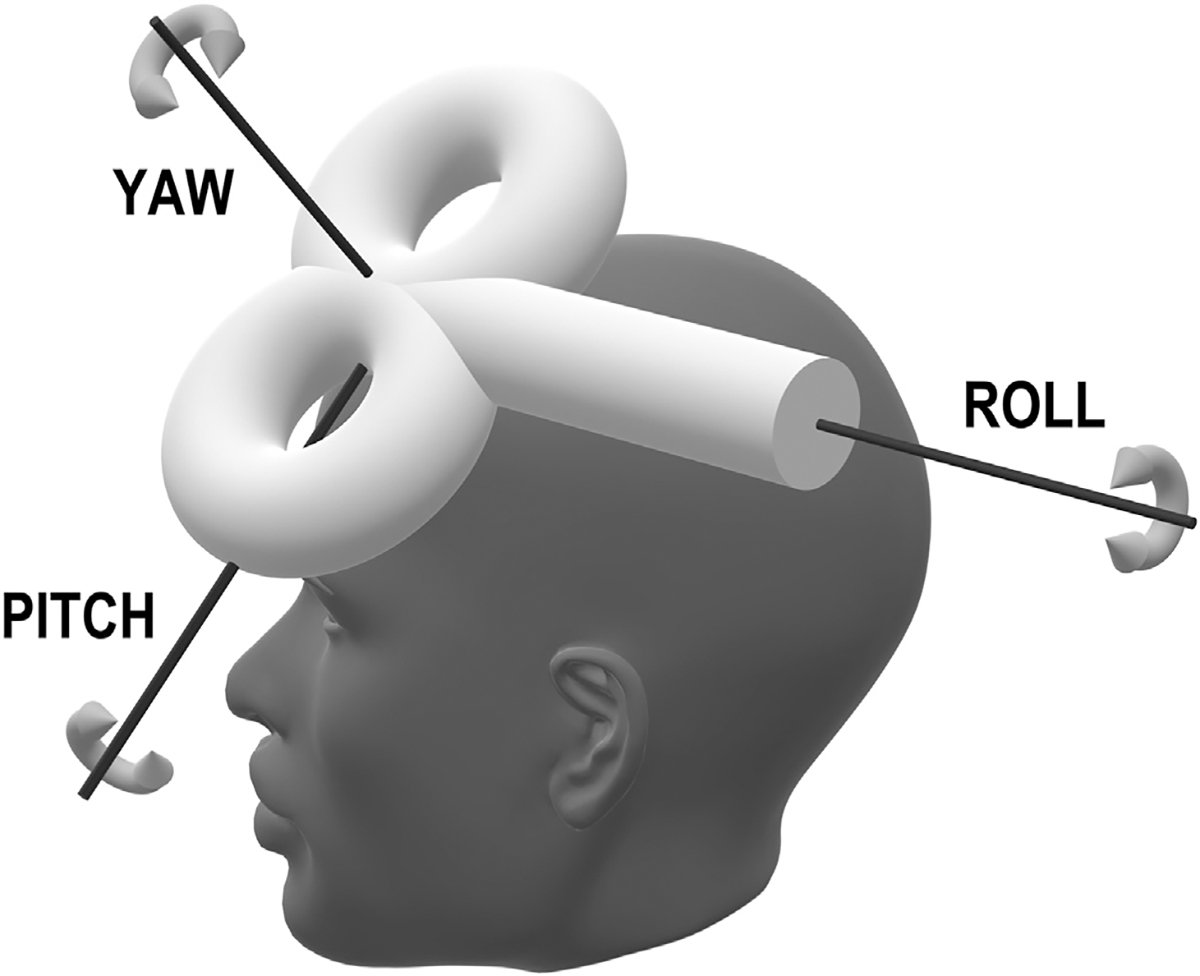
Figure-of-eight transcranial magnetic stimulation coil orientation. The center of the coil face, where the 2 loops meet, is placed on the participant’s scalp over the intended stimulation site. Axes of rotation indicate pitch, roll, and yaw. Pitch and roll are adjusted so that the coil face is tangential to the scalp. The direction of the current induced in the head is determined by coil yaw.

**Figure 2.**
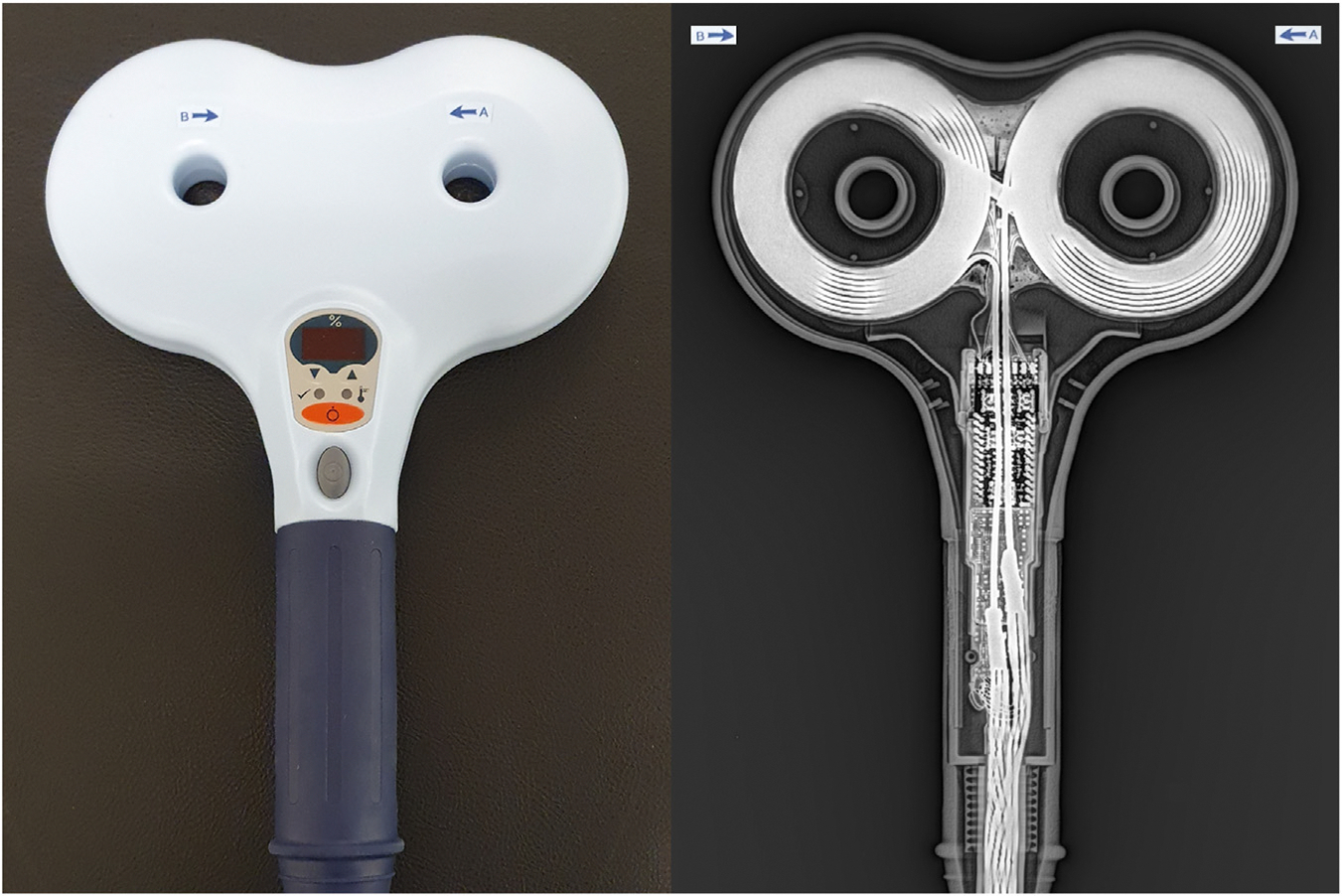
Figure-of-eight transcranial magnetic stimulation coil. (Left panel) Flat figure-of-eight transcranial magnetic stimulation coil. Arrows indicate the initial current direction for this coil (Magstim D70 Remote Coil). Photograph shows operator view of coil, i.e., coil face oriented away from camera. (Right panel) Compound x-ray image of the same transcranial magnetic stimulation coil showing coil windings. Coil face oriented toward x-ray source.

**Figure 3.**
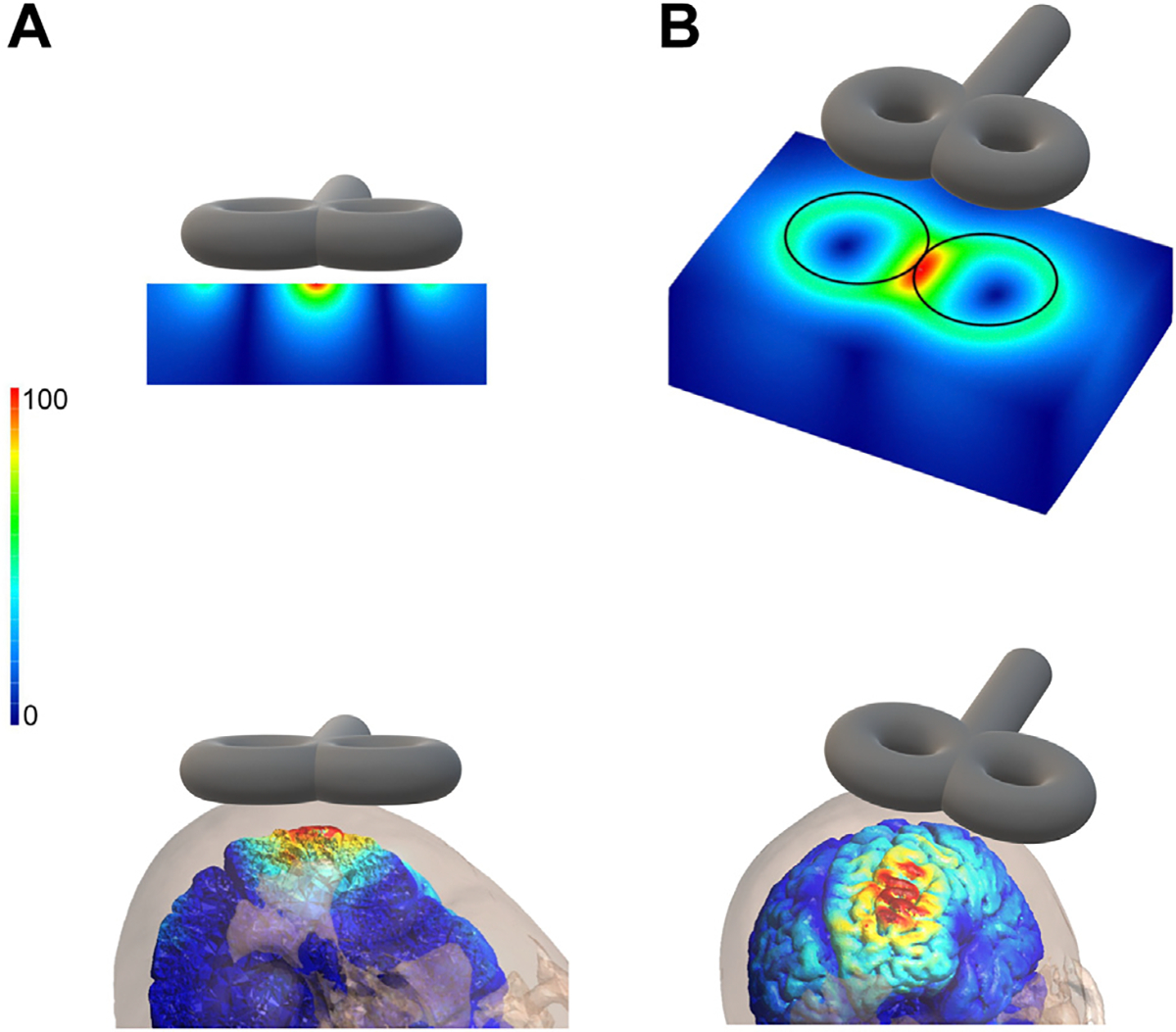
Illustrative electric field magnitude induced by figure-of-eight transcranial magnetic stimulation coil in **(A)** cross-sectional and **(B)** surface perspectives, in slab volumes with homogeneous electrical conductivity (top) and brain tissues (bottom). [Slab volume images modified with permission from Ueno *et al*. (82) and brain tissues modeled in SimNIBS (74).]

**Figure 4.**
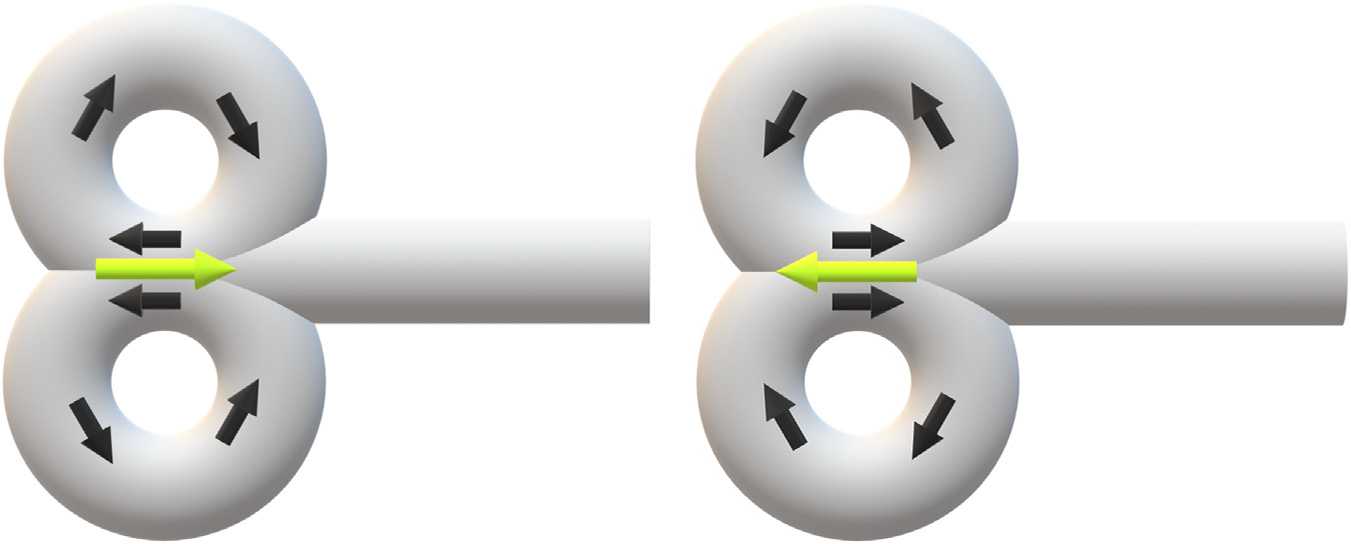
Current direction. Figure-of-eight transcranial magnetic stimulation coil initial current direction (black) induces oppositely directed initial current in the cortex (lime). Note that figure-of-eight coil current direction is not standardized among manufacturers.

**Figure 5.**
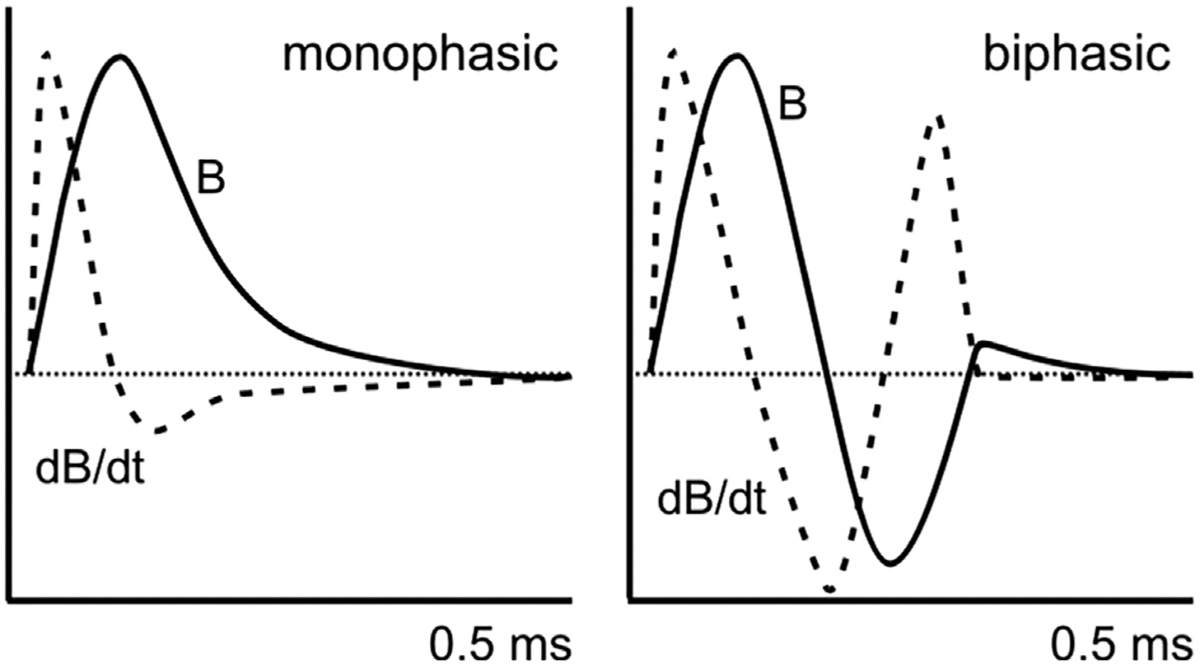
Monophasic and biphasic transcranial magnetic stimulation pulse waveforms. Magnetic-field strength (B, solid line) and its rate of change (dB/dt, dotted line) for monophasic and biphasic stimulus waveforms. dB/dt matches induced electric field waveform. [Modified with permission from Funke (83).]

**Figure 6.**
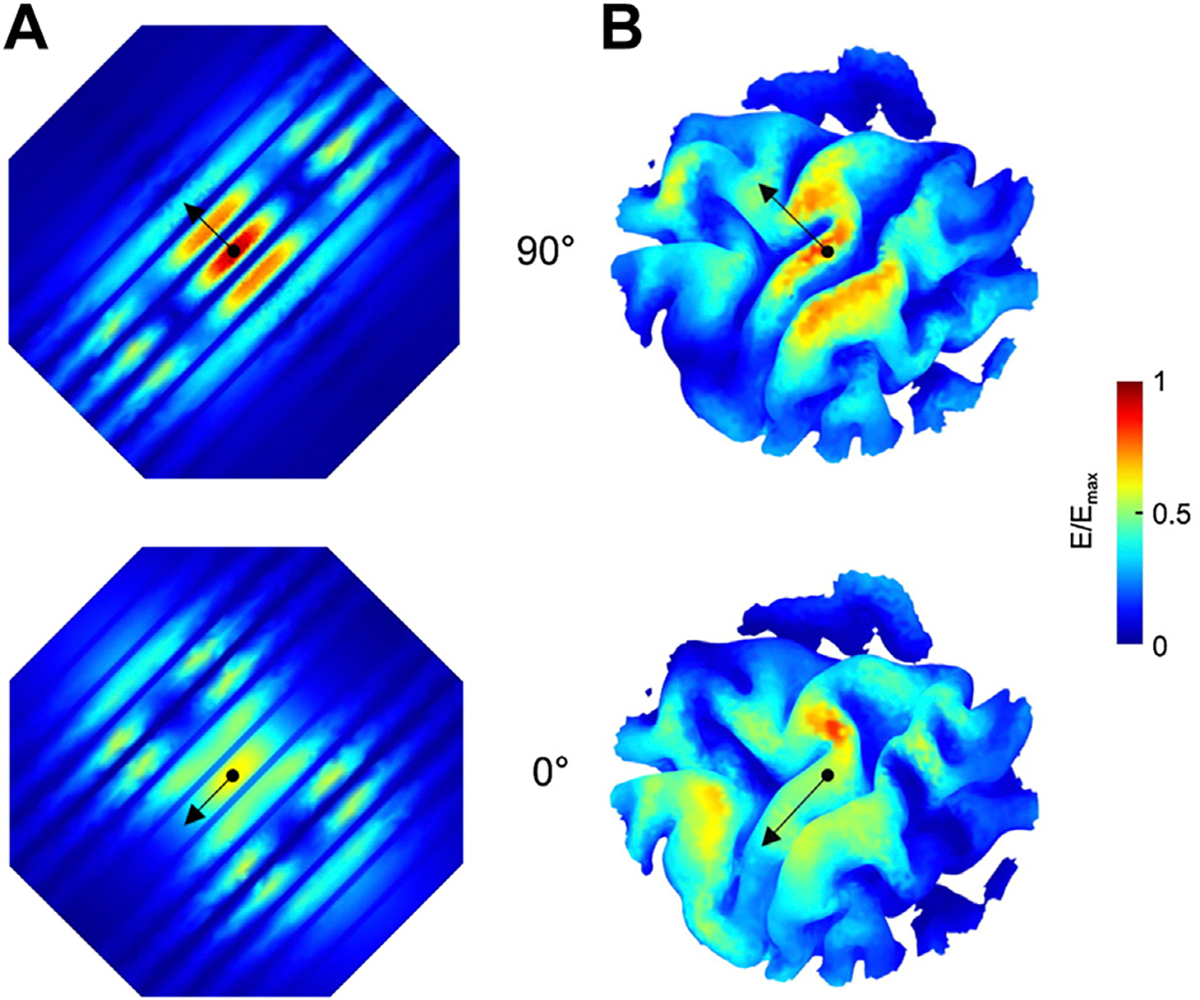
E-field distributions. Electric field magnitude in **(A)** stereotyped straight gyri set 20 mm apart and **(B)** naturalistic left primary motor cortex (centered over hand knob). Blue to red color scale illustrates electric field magnitude as a proportion of maximum (red) value. Transcranial magnetic stimulation coil center indicated by black dot. Current direction indicated by black arrow, being perpendicular (top row) and parallel (bottom row) to local gyri. Length of black arrows scaled to approximately 40 mm. Note the differing sites of maximal electric field magnitude in **(B).** [Modified with permission from Thielscher *et al*. (21).]

Interestingly, current direction is not standardized among TMS equipment manufacturers. Where the coil handle runs parallel to the short axis of the coil (a common design, depicted in Figures 1 and 2), different TMS systems can have opposite current directions. The 180° difference may impact rTMS recruitment of neural populations, particularly when rTMS is applied at intensities near or below motor threshold (63), perhaps influencing plasticity response as per Sommer *et al*. (59). We note that differing response to opposite current direction is not accounted for by E-field techniques that model oppositely directed currents over the same target as symmetrical, i.e., as eliciting equivalent E-field strengths in cortical tissue (21). This is a major short-coming of focusing on E-field strength as a measure of cortical activation and hence dose. One method to address this includes multiscale modeling with morphologically realistic models of neural cell types (4).

## IMPLICATIONS AND OPPORTUNITIES

Coil orientation may impact TMS dosing, activation site, and brain network targeting. Given that these parameters are considered crucial in rTMS treatment, exploring whether therapeutic rTMS coil orientations are optimal seems justified. Several generalized and individualized approaches are canvassed below, each varying in complexity and resource requirements.

### Optimizing Coil Orientation: Generalized Approaches

#### Using Typical Anatomy.

One generalized approach to coil orientation, informed by the interaction between gyral anatomy and E-field strength, may be to orient the coil to direct current flow perpendicular to the main sulcal/gyral axes in the target region. For example, in the left DLPFC, the coil would be rotated clockwise from the current standard orientations to bring the short axis of the coil approximately perpendicular to the superior and inferior frontal sulci that flank the middle frontal gyrus of the DLPFC in an attempt to maximize the E-field magnitude in neural tissue (Figure 8). This perpendicular approach to coil orientation may be supported by research findings in rTMS pain therapy. Andre-Obadia *et al*. (64) found that motor cortex rTMS directed approximately perpendicular to the central sulcus using a posteroanterior coil orientation was effective in alleviating neuropathic pain, while rTMS using a nonperpendicular lateromedial coil orientation was not. Although the research used a relatively small sample (*n* = 16), the single session of perpendicular rTMS reduced average visual numerical scale pain scores by 14% for the whole of the week following treatment. Importantly, the crossover study demonstrated that perpendicular rTMS had superior efficacy to sham rTMS while nonperpendicular rTMS did not. Other experimental work on fibromyalgia by Tzabazis *et al*. (62) centered a 4-coil array over the interhemispheric fissure to examine the efficacy of rTMS in reducing experimentally induced pain in 16 healthy volunteers. The researchers found that directing rTMS current perpendicular to the interhemispheric fissure produced analgesic effects while directing current parallel to the fissure did not. Based on this finding, 20 sessions of perpendicular rTMS were used to treat 16 patients with fibromyalgia and significantly attenuated pain scores for the 4 weeks following treatment. These clinical reports should be interpreted with caution, given the smaller sample sizes and use of experimental coils, but they nevertheless appear to suggest that coil orientation may be a relevant parameter in therapeutic rTMS.

Although directing current flow perpendicular to the main sulcal/gyral axes in the target region could be useful in improving clinical outcomes, there are potential issues with using typical anatomy to guide coil orientation that should be considered. Firstly, when compared with treatment sites in the motor cortex or along the interhemispheric fissure, the major sulcal anatomy is less consistent in rTMS treatment locations such as the DLPFC (65), and the current scalp measurement–based methods for identifying the ideal DLPFC stimulation site localize to heterogeneous regions of the middle frontal gyrus (66). Here, if the treatment target is somewhat equidistant from the superior and inferior frontal sulci, local and more individually variable gyral anatomy may have a greater influence on the cortical E-field. If the gyral crests at the treatment site are not perpendicular to the orientation of the TMS coil, the effective E-field magnitude or dose may be lower than intended. The potential for this local influence is illustrated by E-field modeling of coil orientation in the neighboring inferior frontal gyrus, where lateromedial (i.e., more perpendicular to the major sulci) stimulating current reduced maximal E-field magnitude, perhaps because lateromedial current may run parallel to intermediate sulci within the inferior frontal gyrus. Secondly, in this orientation, the wings of the coil may be closer to the eye and the motor cortex, possibly causing undesired peripheral or cortical stimulation (51). Nevertheless, orienting the TMS coil perpendicular to typical major sulci near the treatment target is a simple and inexpensive adjustment that may warrant clinical investigation because it is supported by therapeutic studies (62,64) and by the nature of the E-field’s interaction with head tissues (21).

#### Using Typical Networks.

**Figure 7.**
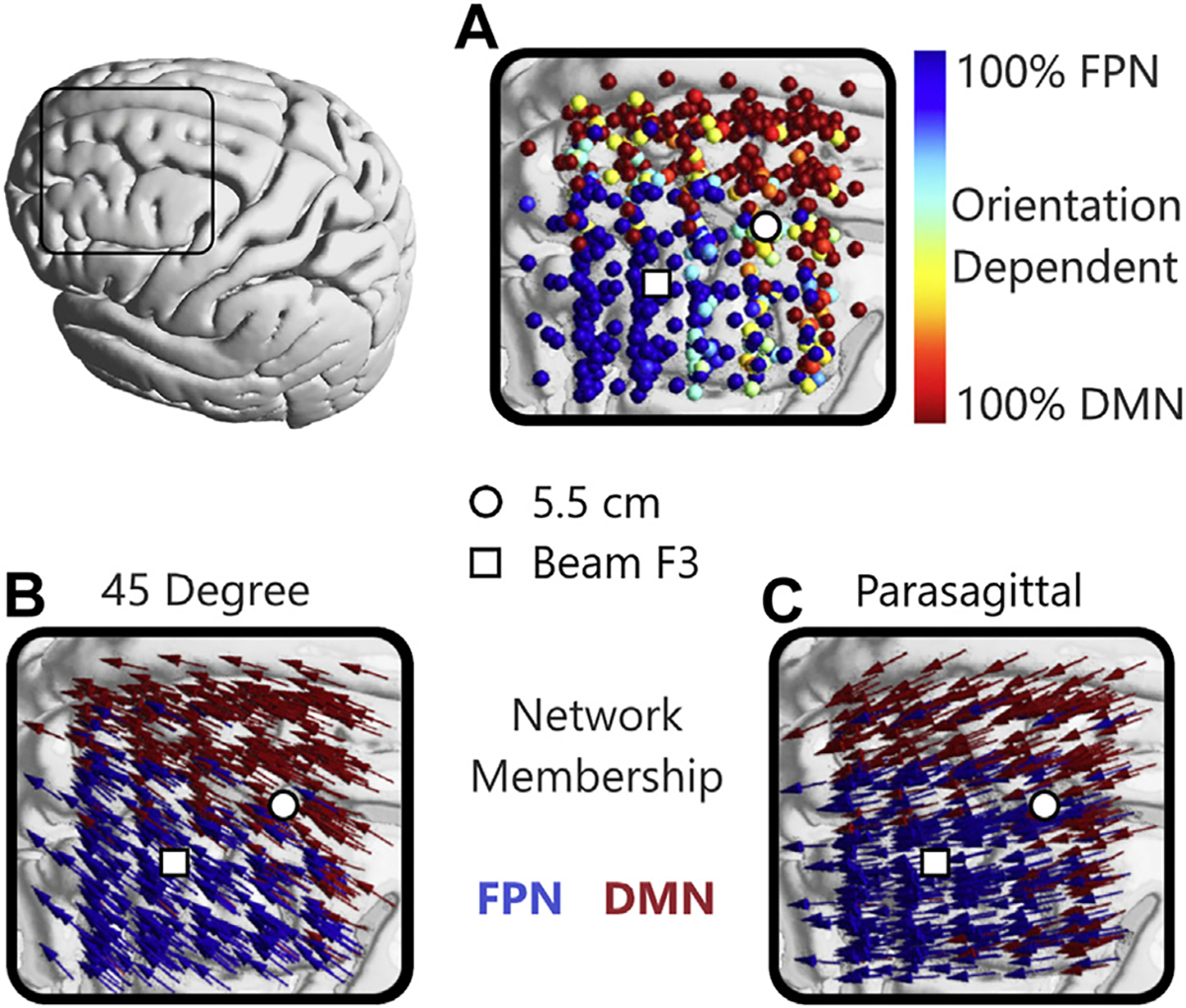
Orientation-dependent network activation in the dorsolateral prefrontal cortex. **(A)** Color of each marker indicates the proportion of orientations activating the frontoparietal network (FPN) or default mode network (DMN) at that location, showing greater orientation dependence in yellow-turquoise. With the 2 coil orientations commonly used in transcranial magnetic stimulation depression treatment, a **(B)** 45° orientation activates the FPN across a smaller range of coil positions than a **(C)** parasagittal orientation. White circle and square indicate approximate 5.5 cm and adjusted Beam F3 sites, respectively, per Trapp *et al*. (56). [Adapted with permission from Opitz *et al*. (51).]

**Figure 8.**
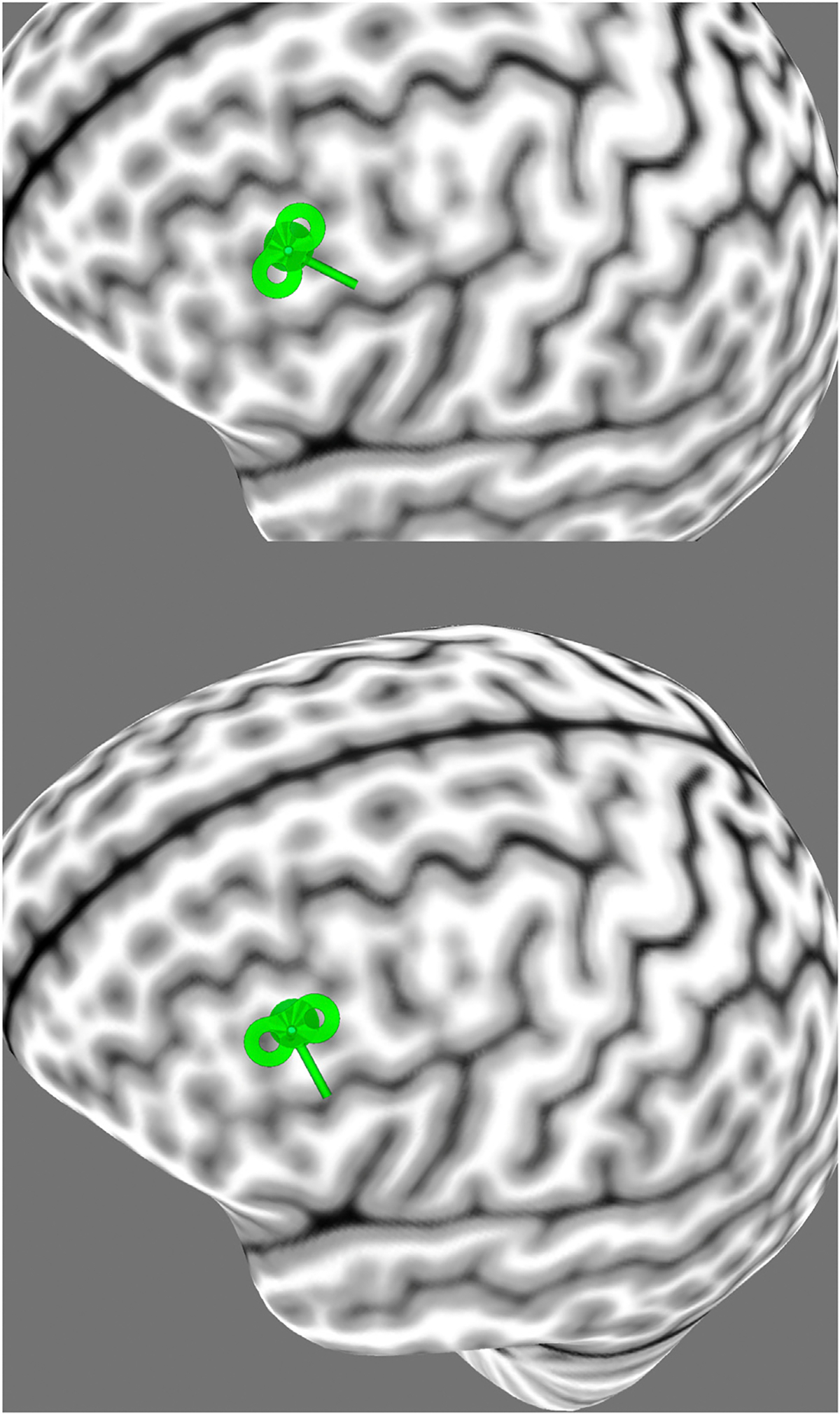
Coil orientation relative to typical major sulci. Coil orientation over a treatment target in the dorsolateral prefrontal cortex. Coil oriented at approximately 45 to the midsagittal plane (top) and perpendicular to superior and inferior frontal sulci flanking the middle frontal gyrus (bottom). (Image produced with Brainsight [Rogue Research].)

The combination of E-field modeling and brain network models may support another generalized approach to rTMS coil orientation in depression treatment. Computational research suggests that the optimal coil orientations for targeting specific networks differ across treatment target locations in the DLPFC (48). Posteromedial sites preferentially target the DMN while anterolateral sites tend to target the FPN and other task-positive networks. However, in the region between these 2 sites, network targeting appears to be influenced by coil orientation, i.e., parasagittal current preferentially targets the FPN, while 45° current preferentially targets the DMN (48). Interestingly, these 2 coil orientations are somewhat similar to the 2 most common orientations used in clinical practice for depression. These findings suggest that selecting the coil orientation that is optimized for the chosen network target may provide a greater margin of error in coil localization. For example, if clinicians wanted to influence the FPN but coil placement was not optimal (i.e., perhaps not sufficiently anterolateral), it is possible that a parasagittal coil orientation would still target the FPN even if the coil was erroneously positioned in the orientation-dependent transition zone (Figure 7). Although this work focuses on the DLPFC, network targeting is being investigated in rTMS for neuropsychiatric disorders other than depression that involve different brain networks, which may support a network-informed approach to coil orientation in other brain regions. While potentially useful, we note that these studies test for hypothetical targeting of different networks and do not directly measure network engagement. To test this hypothesis definitively, it would be necessary to demonstrate differential changes in neurophysiological, neuroimaging, or clinical outcomes as a function of coil orientation utilizing individualized functional connectivity.

### Optimizing Coil Orientation: Individualized Approaches

#### Anatomical Approach.

**Figure 9.**
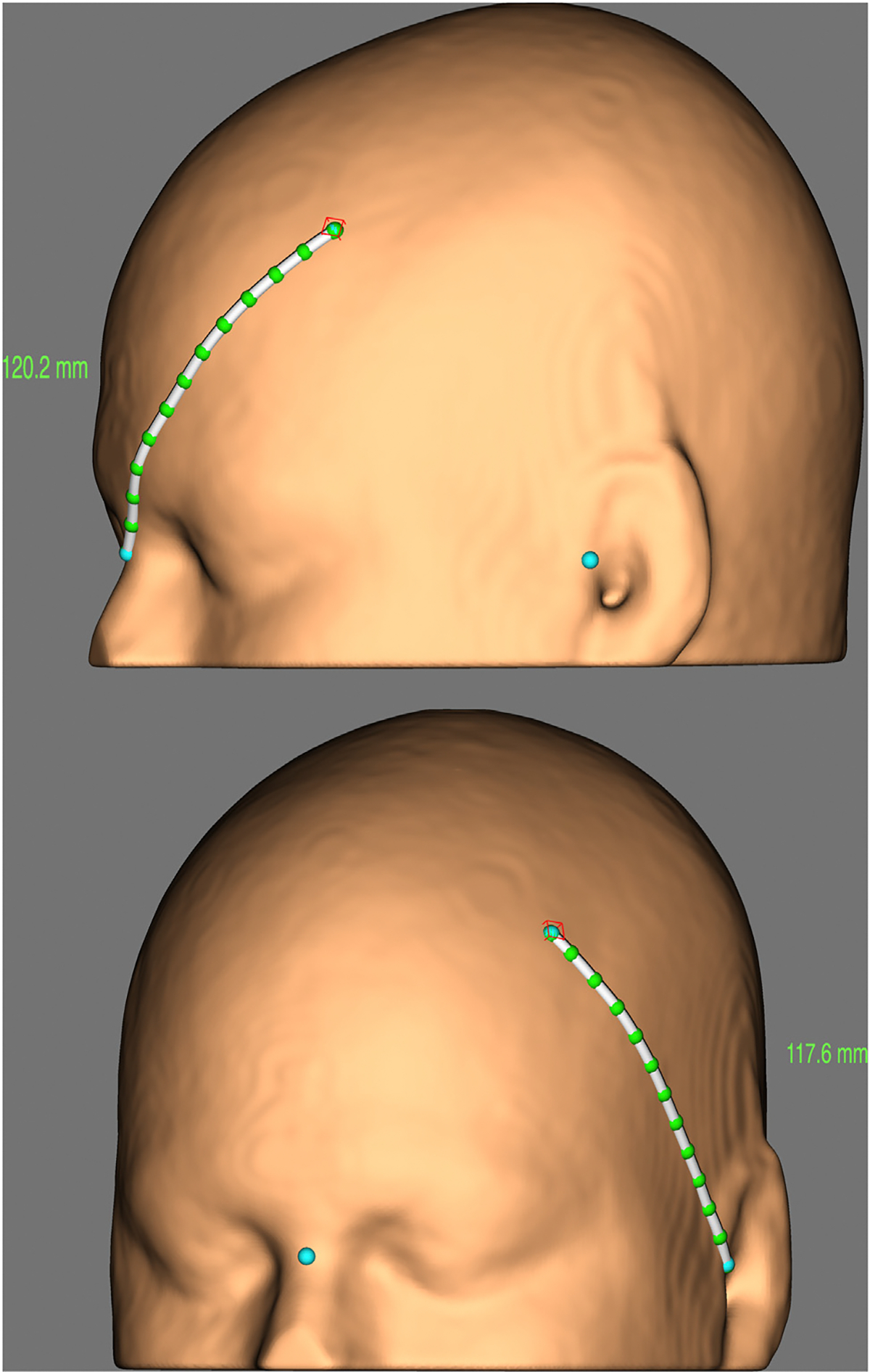
Illustration of in silico scalp measurement. Scalp measurements taken along the reconstructed skin surface. A point on the scalp (red cube) and scalp landmarks (blue spheres at nasion and left preauricular notch) are marked within a software reconstruction of patient magnetic resonance imaging data. Measurements extracted in silico can be used to locate scalp points in vivo without requiring a neuronavigation system. One point would mark coil position, and another point (perhaps along the axis of the coil handle) would mark coil orientation. [Image produced with Brainsight ruler tool (Rogue Research) to illustrate the techniques proposed by Vaghefi *et al*. (70).]

Individualized approaches to coil orientation are possible but require person-specific imaging and a method of coregistering image and scalp locations. The latter can be achieved to within 5 mm of a defined target due to the high correspondence between measurements made on an individual’s scalp and measurements extracted from their imaging data (67) (see Figure 9 for illustration), and even greater precision can be achieved with stereotaxic neuronavigation systems. Where neuronavigation is unavailable, another pair of in silico scalp measurements could be used to define the coil orientation by indicating a position along the axis of the coil handle. At its simplest, a treatment site may be identified based on distance from motor cortex hand representation or a specific scalp location determined by proportional calculations that account for differences in head size (68). Once identified, the target could be located on participant-specific imaging to gain a visual appreciation of the surrounding gyral anatomy. Then the coil could be oriented perpendicular to the local sulci present in that individual’s unique cortical folding patterns in an attempt to maximize the E-field induced at the target site. In circumstances where E-field modeling is unavailable, this estimation-based approach may go some way toward maximizing the E-field in the cortical target and minimizing any lateral displacement between the selected coil position and the site of cortical stimulation.

#### Anatomy and E-Field Modeling.

A more comprehensive individualized approach would be to load individuals’ MRI scans into E-field modeling software that can determine the coil position and orientation that elicits the maximum E-field at the chosen target site. E-field modeling has recently been incorporated in stereotaxic TMS neuronavigation systems (e.g., Brainsight, Rogue Research; SmartFocus, Nexstim), and it is now possible for TMS operators to quantify (and visualize) modeled E-fields and their relationships with coil orientation. The scans, modeling resources, and neuronavigation equipment represent additional costs, although scalp measurement techniques may substitute for the neuronavigation equipment (see Figure 9). It is important to note that field modeling adds at least some complexity to standard clinical practice (69), which may make it more challenging and expensive to implement in some clinical settings.

#### Anatomy, Functional Connectivity, and E-Field Modeling.

The question of optimal coil orientation has been partly addressed via novel targeting strategies that integrate individualized anatomy, E-field modeling, and network connectivity profiles assessed via functional MRI (49,70). This approach may be clinically useful because targeting specific brain networks appears to be important for clinical response to rTMS (71,72), and there is some evidence that rTMS targeted to different brain networks is associated with clinical improvement in distinct symptom clusters (73). However, it is unclear how selective this targeting needs to be, i.e., whether concurrent targeting of other networks might reduce therapeutic efficacy or symptom specificity. In response, these recent techniques determine the coil orientation (and location) that is expected to maximize stimulation of the chosen network while avoiding nontarget networks (49,70). Although these targeting strategies have not been tested yet in clinical rTMS, they do highlight the possibility that TMS coil orientation may be an underoptimized treatment parameter.

The technical and computational demands inherent in E-field modeling and network-based approaches to coil orientation and positioning may initially hinder clinical implementation. However, more user-friendly implementations of E-field modeling continue to evolve (36,74–76), and their integration with neuronavigation systems reduces, but does not eliminate, the technical challenge. A potential solution may be to develop remote or online services for processing functional MRI data and identifying optimal treatment parameters. If such a service were available, clinicians could upload a patient’s scan and be provided with an optimized coil orientation, localization, and stimulation intensity for applying individualized rTMS treatment.

## LIMITATIONS

This discussion of the effect of cortical morphology on TMS-induced E-fields has only considered current direction induced by figure-of-eight TMS coils. Other coil types, e.g., H-coils, induce less focal E-fields (77), perhaps reducing the need for accurate coil placement while sacrificing selectivity but still induce directed currents that will interact with head tissues as described above. Although not detailed here, experimental coils have recently been developed that are able to stimulate in multiple current directions (78). Interestingly, multidirectional, or rotational, stimulation may be a promising approach to reducing orientation-dependent variability in neural activation. Rotational stimulation has been shown to evoke larger amplitude motor responses than unidirectional stimulation (78), perhaps by activating a wider range of neural populations, thus underscoring the interaction between gyral anatomy and coil orientation.

Furthermore, our focused considerations of coil orientation, targeting, and intensity must be considered together with the numerous TMS parameters that may determine the directionality (i.e., excitatory or inhibitory) and occurrence of synaptic plasticity changes that subserve network modulation (79,80). It is also important to note that we have primarily considered the interaction between current direction and macroanatomy and only briefly highlighted potentially important interactions between current direction and microanatomy [see modeling in Aberra *et al*. (4) and Shirinpour *et al*. (81)]. Finally, we restate that E-field modeling is a theory-driven computational process. Although the models have been validated in the motor cortex (21,26) they are necessarily incomplete, and it is possible that they overestimate the influence of coil orientation on cortical stimulation and network targeting.

## CONCLUSIONS

The evidence reviewed herein suggests that coil orientation deserves consideration as an optimizable parameter when applying rTMS to the cerebral cortex. In this review, we summarized the impact that TMS-induced current direction and local gyral anatomy have on cortical activation and how this interaction can impact TMS dosing, the site of TMS activation, and TMS network engagement. These parameters are of considerable interest for the roles that they play individually and collectively in influencing rTMS’s therapeutic effects. Based on our review of the evidence accumulated to date, we have suggested several ways in which TMS coil orientation could be optimized at an individual or population level with currently available tools.
